# Supplementary material for: A Novel Mitochondrial Genome Fragmentation Pattern in the Buffalo Louse Haematopinus tuberculatus (Psocodea: Haematopinidae)
Source: Int J Mol Sci. 2022 Oct 28;23(21):13092. doi: 10.3390/ijms232113092 (PMC9658350; doi:10.3390/ijms232113092)

Supplementary Materials

Table S1

PCR primers used to amplify and sequence the mitochondrial genome of the buffalo louse, *Haematopinus tuberculatus.*

Primer	Sequence (5' to 3')	Minichromosome
*nad*4F	TATTGGGGTTGTGGGTGCTTGTT	K-*nad*4-*atp*8-*atp*6-N
*nad*4R	AACAAGCACCCACAACCCCAATA	K-*nad*4-*atp*8-*atp*6-N
*cyt*bF	TCCTCCACACATCCAACCTGAAT	E-*cyt*b-V
*cyt*bR	ATTCAGGTTGGATGTGTGGAGGA	E-*cyt*b-V
*cox*1F	TTACCTGGATTTGGCTTGATTTC	*nad*2-I-*cox*1-L_2
*cox*1R	GAGAAATCAAGCCAAATCCAGGT	*nad*2-I-*cox*1-L_2
*cox*2F	GAGACGCAATTCCTGGACGACTTAA	D-Y-*cox*2-S_1-S_2-P-*cox*3-A
*cox*2R	AGAAGGGATAGTCCAAGAATGAATG	D-Y-*cox*2-S_1-S_2-P-*cox*3-A
*nad*1F	AAGTAGTTGCTAAGGTTGGGT	Q(−)-*nad*1(−)-T(−)-G-*nad*3-W
*nad*1R	ACTAATAGCCCTTCCTAACAT	Q(−)-*nad*1(−)-T(−)-G-*nad*3-W
*nad*4LF	TGGTATTAGAGGGAAGTTTAGGG	R-*nad*4L
*nad*4LR	AGTCAACCCTAAACTTCCCTCTA	R-*nad*4L
*nad*5F	GTATTACAGCCTTAGGGGAGT	H-*nad*5-F-*nad*6
*nad*5R	AGGTAATCAAGAACAGAAAGG	H-*nad*5-F-*nad*6
*rrn*SF	AGTCTGGATAAGTCGTAACAAAG	*rrn*S-C
*rrn*SR	CTTTGTTACGACTTATCCAGACT	*rrn*S-C
*rrn*LF	GAATGAACGGTCTAACAAAAGTA	L_1-*rrn*L
*rrn*LR	ATCACAAGCATTGAGCAGGTCGT	L_1-*rrn*L
MF	ACCACAGAAATAAAGGAAGAAGG	M
MR	ATCCTTCTTCCTTTATTTCTGTG	M

Figure S1 Numbers of fragmented mitochondrial minichromosomes of nine sucking lice with at least one unidentified gene in their mt genomes.

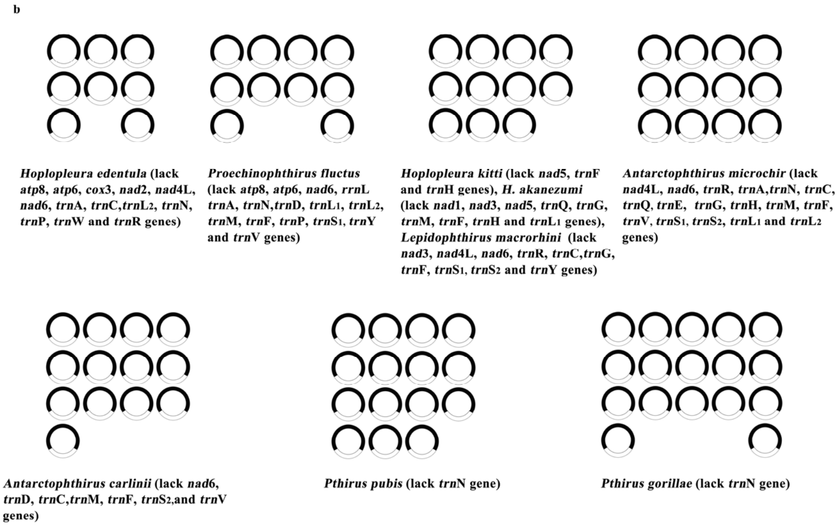

Figure S2 PCR verification of the ten mt minichromosomes of the buffalo louse, *Haematopinus tuberculatus*. Lane M: DL5000 DNA marker; Lane 1–10: *trn*K-*nad*4-*atp*8-*atp*6-*trn*N, *trn*E-*cyt*b-*trn*V, *nad*2-*trn*I-*cox*1-*trn*L_2, *trn*D-*trn*Y-*cox*2-*trn*S_1-*trn*S_2-*trn*P-*cox*3-*trn*A, *trn*Q (−) -*nad*1 (−) -*trn*T (−) -*trn*G-*nad*3-*trn*W, *trn*R-*nad*4L, *trn*H-*nad*5-*trn*F-*nad*6, *rrn*S-*trn*C, *trn*L_1-*rrn*L, *trn*M.

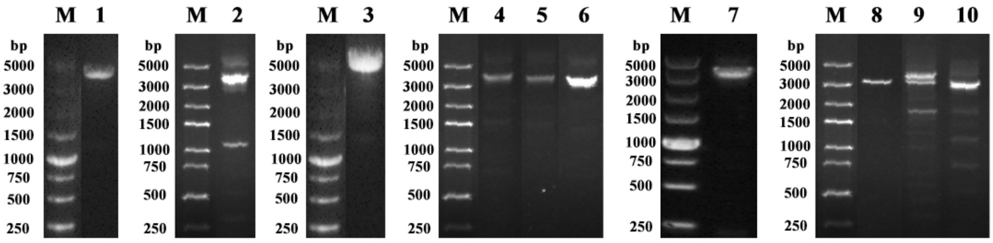

Supplement: Supplementary file 1 [file ijms-23-13092-s001.zip › ijms-1906564-supplementary.pdf]
